# Temperature-dependent development and reproduction of rice leaffolder, *Marasmia exigua* (Lepidoptera: Pyralidae)

**DOI:** 10.1371/journal.pone.0187972

**Published:** 2017-11-10

**Authors:** Qiu-Ju Liao, Ya-Jun Yang, Jia Wang, Xiao Pang, Chun-Mei Xu, Cheng-Lin Peng, Zhong-Xian Lu, Ying-Hong Liu

**Affiliations:** 1 Institute of Entomology, College of Plant Protection, Southwest University, Chongqing, China; 2 State Key Laboratory Breeding Base for Zhejiang Sustainable Pest and Disease Control, Institute of Plant Protection and Microbiology, Zhejiang Academy of Agriculture Sciences, Hangzhou, China; 3 Qianwei Plant Protection Station, Leshan, China; USDA Agricultural Research Service, UNITED STATES

## Abstract

*Marasmia exigua* (Butler) (Lepidoptera: Pyralidae) is one of the major rice leaffolders negatively affecting the rice production in the world. The growth and development of *M*. *exigua* was studied at seven constant temperatures (10, 15, 20, 25, 27, 30 and 35°C). The results showed that *M*. *exigua* eggs failed to hatch at 10°C and the larvae could not complete development at 15 and 35°C. The developmental times of each stage, survival rates of pre-adult, adult longevity, fecundities and oviposition days of *M*. *exigua* at 20, 25, 27 and 30°C were investigated using age-stage, two-sex life table. The total pre-adult development time decreased with the increase in temperature decreasing from 61.58 days at 20°C to 28.94 days at 30°C. The highest survival rate was observed at 25°C (73%). Male adult longevities were generally longer than that of females, except at 30°C. The highest mean fecundity, age-stage specific fecundity and age-specific fecundity peak values were all observed at 27°C. The maximum intrinsic rate of increase *r* and finite rate of increase *λ* were observed at 27°C, while the maximum net reproduction rate *R*_*0*_ was observed at 25°C. The longest mean generation time occurred at 20°C and the shortest at 27°C. These results provide better understanding on the development, reproduction and dynamic of *M*. *exigua* populations, their distribution, and might be utilized to forecast and manage *M*. *exigua* outbreaks in China.

## Introduction

Rice (*Oryza sativa* L.) is one of the most important food and is a crucial staple for more than half the world’s population [1]. Rice leaffolders, a group of Lepidopteran pests, are increasingly detrimental to rice production in China, due to changes in cultural practices and the use of high-yield varieties [2]. In addition, the overuse of nitrogen fertilizers has also contributed to the outbreaks [3–5]. These pests are widely distributed in the rice growing regions of tropical and temperate areas of Asia, Oceania, and Africa. At least three species of rice leaffolders, *Cnaphalocrocis medinalis* (Guenée), *Marasmia exigua* (Butler), and *M*. *patnalis* (Bradley) have gained major pest status in some rice production zones [6]. Due to similar habits and morphological characteristics, *M*. *exigua* is often mis-identified as a different species of leaffolder by researchers in many countries [6–8]. In China, *M*. *exigua* has been mistaken for *C*. *medinalis* [9, 10]. Over the past few decades, *M*. *exigua* has drawn much attention for its severe damage to rice in some places in China. In general, *M*. *exigua* larvae fold leaves and scrapes off the green mesophyll tissue causing reductions in leaf photosynthetic activity and crop yields [11]. It has been shown that yield loss could reach 28.5–29.7% when leaf blade damages were 41.2–42.8% [10]. However, less research emphasis has been placed on *M*. *exigua* compared to *C*. *medinalis*. In the last few years, *M*. *exigua* has dominated paddy fields in many places of Sichuan Basin in southwest China raising concerns that it might become a principal rice pest.

With the increasing awareness of problems associated with pesticide abuse and the rising popularity of organic farming, the development of safe and ecological control strategies have become an important goal in pest management [12]. Thus understanding the ecology of a specific pest is a prerequisite for the subsequent formulation of management strategies. Although morphological characteristics and the biology of *M*. *exigua* have been described [6, 7, 9, 10, 13], information on its ecology remains scarce and the impact of temperature on its development and fecundity, and population parameters are unknown. Temperature is an important abiotic factor affecting the life activities of insects [14]. Ecological effects on an insect’s life history, population dynamics, geographical distribution and management strategies can be further understood through temperature-dependent experiments [15]. Life table analyses are a means for determining population characteristics that project population growth, describe developmental characteristics such as survival rates, reproduction rates, and life expectancies in a pest population [16–19]. The traditional age-specific and time-specific life tables that are usually used in female populations, ignore the variable developmental rates of individuals and are unable to calculate the survival rates of immature stages and additional important parameters including the intrinsic rate of increase *r*, and finite rate of increase *λ* [20–24]. The age-stage, two-sex life table, which not only takes into account both female and male populations, as well as the stage differentiations among individuals and calculates the survival rates of all stages and important population parameters, was used to study *M*. *exigua* life history at different constant temperatures [25, 26]. In addition, the lower developmental threshold and thermal constant for each stage were calculated using a linear model.

The objective of this study is to understand the biological and ecological characteristics of *M*. *exigua* for forecasting population growth and estimating geographical distributions.

## Materials and methods

### Insect collection

The overwintering *M*. *exigua* pupae were collected from rice stubble fields in Qianwei county (Leshan, Sichuan Province, China; E103°93’, N29°21’) in March 2016 and reared in an incubator (LAC-250HPY-2, Shanghai Longyue Instruments, Shanghai, China) under constant conditions (26±1°C, 80±5% RH, and a photoperiod of 14L:10D) for two generations. The pupae were placed in cups with moist cotton in the bottom to maintain high humidity and the rims were covered with gauze secured with rubber bands. Upon adult emergence, the *M*. *exigua* paired adults were transferred to an oviposition container with 3–5 pairs per cup (8 cm in diameter, 10 cm in height). The containers with paired adults were covered with plastic film punctured with small holes for ventilation. Sterilized cotton balls soaked with 10% honey solution were hung from the top to serve as food for the adults. Eggs were collected daily. Larvae were fed fresh rice leaves that were daily until pupation. Insect were collected from our experimental field. *M*. *exigua* is a pest species and is neither endangered nor protected.

### Developmental times, fecundities and longevities

*M*. *exigua* eggs were collected on the same day they were laid and placed into disposable petri dishes (12-cm-diameter) with a moist filter paper. The dishes were then placed into incubators set at seven constant temperatures (10, 15, 20, 25, 27, 30, and 35°C), maintained with relative humidity 80±5% and a photoperiod of 14L:10D. At egg hatch, the neonate larvae were transferred individually to a 9-cm-diameter disposable petri dish using a banister brush. Ninety-six to three hundred larvae were placed under each temperature condition. The food source was rice leaf cuts (8-cm long) wrapped in moist cotton balls at both ends with a moist filter paper. Both leaf cuts and moist filter papers were replaced every day until pupation. Observations were done daily, and the larval instars were determined at each larval molting using an optical microscope. The survival and developmental stages of each larva were recorded daily. Upon pupation, individual pupae were transferred into glass tubes (1.5cm diameter, 10cm high) sealed with gauze and moist cotton balls in the bottom. Upon emergence the adults were paired and each pair placed in a plastic oviposition container (320mL), covered with perforated plastic film. Sterilized cotton balls saturated with 10% honey solution and replaced daily were provided as food sources. Eggs were collected daily, and the fecundity (the number of eggs produced by per female) and survival were recorded daily until the death of all individuals. The eggs, larvae, prepupal and pupal stages (denoted as pr and p), pre-adult times (the period counted from egg to pupal stage), pre-adult survival rates (pupa number/egg number), adults longevities, total lifespans, total pre-oviposition periods (TPOP, the period counted from egg to first oviposition), adult pre-oviposition periods (APOP, the period counted from adult emergence to first oviposition), fecundities (the mean number of eggs produced in a female’s lifetime) and oviposition days were calculated based on the experimental data.

### Life table analysis

The data for development time and *M*. *exigua* egg hatchability were analyzed using IBM SPSS Statistic (Version 22.0), the mean values were compared by one-way analysis of variance followed by Dunnett T3 test at a significance of 0.05. Experimental life table data obtained at different temperatures were analyzed through the age-stage, two-sex life table analysis program TWOSEX-MS Chart (Version 2016.06.02) available from http://nhsbig.inhs.uiuc.edu/wes/chi.html [25, 26]. The age-stage specific survival rates (*S*_xj_), defined as the probability that a new-laid egg will survive to age *x* and stage *j*, age-stage specific fecundities (*f*_*x*8_) which are the mean egg number produced by *M*. *exigua* female of age *x* (8 is the female stage), age-specific survival rate (*l*_*x*_) (the probability that a new-laid egg will survival to age *x*) and age-specific fecundity (*m*_*x*_) (the number of eggs that an individual will produce at age *x*) were calculated and graphed. The graphs were created using Sigmaplot 12.0. The mathematical relationships were expressed by the following formulas:

$$l_{x}=\sum_{j=1}^{m}S_{xj}$$ (1)

$$m_{x}=\frac{\sum_{j=1}^{m}S_{xj}f_{xj}}{l_{x}}$$ (2)

(m is the stage number) [26].

Based on this, we calculate the population parameters *r*, *λ*, *R*_*0*_ and *T*. The intrinsic rate of increase *r* was estimated using the iterative bisection method from Lotka-Euler formula:

$$\sum_{x=0}^{\infty }e^{-r\left(x+1\right)}l_{x}m_{x}=1$$ (3)

with age *x* indexed from 0 [27]. The finite rates of increase *λ*, net reproduction rates *R*_*0*_ and mean generation times *T* (the time that a population needs to increase to *R*_*0*_-fold of its size at the stable age distribution) were estimated by:

$$\lambda =e^{r}$$ (4)

$$R_{0}=\sum_{x=1}^{\infty }l_{x}m_{x}$$ (5)

$$T=\text{ln}R_{0}/r_{m}$$ (6)

The standard errors of general statistics and population parameters (*r*, *λ*, *R*_*0*_, *T*) were calculated by running a Bootstrap [28] in TWOSEX with 100, 000 bootstraps, and the variances were estimated using a pick 1 by 1 method in TWOSEX, which is based on percentile differences and 95% CI of the normalized distribution of differences.

### Developmental thresholds and thermal constants

The mean development rate (1/development time) values were used for fitting linear function of temperature. The lower temperature thresholds (*t*) and thermal constants (*K*) were estimated by an ordinary linear model (Eq 1) for each stage of *M*. *exigua*.

y=a+bx (7)

where *y* is the rate of development at temperature *x*, *a* and *b* are constants, which were estimated by least square regression. The lower developmental threshold (*t*) was calculated from *t* = −*a*/*b*, while the thermal constant (*K*) in degree days (*DD*) was estimated as *K* = 1/*b*. Standard errors (*SE*) for *t* and *K* were calculated from Eqs 8 and 9:

$$SE_{t}=\frac{\bar{y}}{b}\sqrt{\frac{s^{2}}{N{\bar{y}}^{2}}+{\left(\frac{SE_{b}}{b}\right)}^{2}}$$ (8)

$$SE_{K}=\frac{SE_{b}}{b^{2}}$$ (9)

where *s*^2^ is the residual mean square of *y*, $\bar{y}$ is the sample mean, and *N* is the sample size (number of temperatures tested) [29].

## Results

### Developmental times, survival rates and longevities

**Fig 1:**
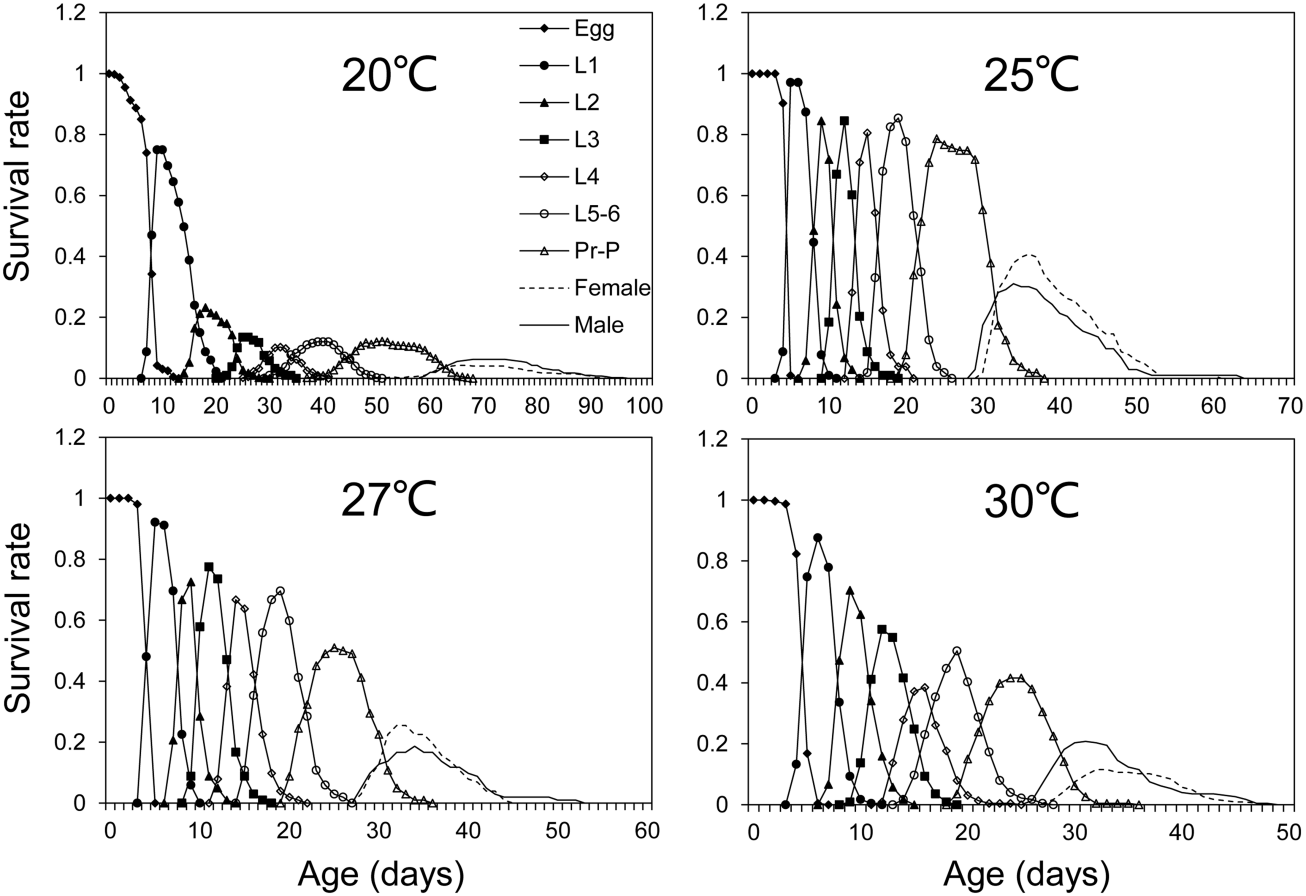
Age-stage specific survival rates (*S*_*xj*_) of *Marasmia exigua* at four temperatures. L_1-6_, larval instar. Pr-P, pre-pupal and pupal stages.

The development of *M*. *exigua* eggs under seven constant temperatures were observed. All eggs failed to hatch at 10°C, some eggs could hatch at 15°C and 35°C but all died at the fifth and first instar, respectively (Table 1). The results showed that *M*. *exigua* could complete its life cycle at four temperatures 20°C, 25°C, 27°C and 30°C. The mean developmental times of eggs were significantly different (*P*< 0.05) except for 25°C and 30°C, with the longest developmental times (21.66 days) at 15°C, and the shortest (4.49 days) at 27°C (Tables 1 and 2). It is noteworthy that some *M*. *exigua* larvae developed into a sixth instar at 20°C, but that is not the case for larvae at 25, 27, and 30°C (Table 2). There were significant differences among pre-adult times at the four temperatures. In general, the mean pre-adult time decreased as the temperature increased within a certain range, from 61.58 days at 20°C to 28.94 days at 30°C (Table 2). The temperature could significantly affect pre-adult survival rates, and the highest survival rate was observed at 25°C (Table 2). The survival rate of the egg to the second instar larval stage fell sharply at 20°C, but declined smoothly and steadily at the other stages and temperatures (Fig 1). The female adult longevities exhibited significant differences except at 25°C and 30°C. The longest to the shortest longevity were at 20°C> 25°C> 30°C>27°C, respectively. The female total lifespans also showed significant differences at the four temperatures, with the longest (79.53d) at 20°C, the shortest (38.81d) at 27°C, and 20°C> 25°C> 30°C> 27°C, respectively. The male adult longevity showed significant differences except at 27°C and 30°C, with the longest to the shortest longevities at 20°C> 25°C> 27°C> 30°C, respectively. Similarly, the male total lifespans decreased with the rise in temperatures, and showed significant differences among the four temperatures (Table 3).

**Table 1:** Hatchability (%) and developmental times (days) of *Marasmia exigua* eggs at different temperatures.

Temperature(°C)	No. egg	Hatchability(%)	Developmental time (days)	
			Mean ± SE	Median
10°C	527	0	—	—
15°C	334	50.60	21.66 ± 0.05a	22
20°C	824	74.12	8.29 ± 0.03b	8
25°C	482	96.89	4.91 ± 0.03c	5
27°C	931	94.09	4.49 ± 0.05d	4
30°C	595	89.08	5.00 ± 0.04c	5
35°C	630	7.91	—	—

SE, standard error. Means (± SE) in a column followed by different letters were significantly different (Dunnett T3 test, *P*< 0.05).

**Table 2:** Developmental times (days) and survival rates (%) of *Marasmia exigua* at four temperatures.

Item	20°C		25°C		27°C		30°C	
	n	$\bar{\text{x}}\;\pm \;\text{SE}$	n	$\bar{\text{x}}\;\pm \;\text{SE}$	n	$\bar{\text{x}}\;\pm \;\text{SE}$	n	$\bar{\text{x}}\;\pm \;\text{SE}$
Egg	300	8.26 ± 0.04a	100	4.91 ± 0.03b	96	4.49 ± 0.05c	200	5.00 ± 0.04b
L_1_	104	8.10 ± 0.12a	94	3.59 ± 0.07b	88	3.56 ± 0.08bc	176	3.39 ± 0.05c
L_2_	61	7.75 ± 0.17a	94	2.68 ± 0.07c	87	2.33 ± 0.07d	166	3.13 ± 0.07b
L_3_	58	5.79 ± 0.25a	93	2.86 ± 0.07c	85	3.45 ± 0.08b	160	3.36 ± 0.09b
L_4_	58	5.10 ± 0.14a	93	3.01 ± 0.08b	79	3.15 ± 0.07b	148	2.49 ± 0.06c
L_5_	32	6.63 ± 0.28a	89	5.07 ± 0.11b	64	5.38 ± 0.11b	118	4.44 ± 0.11c
L_6_	18	6.92 ± 0.25	0	—	0	—	0	—
Pr-P	45	16.89 ± 0.30a	75	9.57 ± 0.06b	49	8.12 ± 0.06c	78	7.46 ± 0.07d
Pre-adult time	45	61.58 ± 0.51a	75	31.63 ± 0.18b	49	30.35 ± 0.26c	78	28.94 ± 0.19d
Pre-adult survival rate	400	11.25 ± 1.57d	103	72.82 ± 4.36a	102	48.04 ± 4.95b	226	34.51 ± 3.16c

L_1-6_, larval instar; Pr-P, pre-pupal and pupal stages; *n*, effective sample size; $\bar{\text{x}}$, mean value; SE, standard error. Means (± SE) in the same row followed by different letters were significantly different by the pick 1 by 1 test based on the CI of difference (*P*< 0.05).

**Table 3:** Adult longevity (days) and lifespan (days) of *Marasmia exigua* at four temperatures.

Item	20°C		25°C		27°C		30°C	
	n	$\bar{\text{x}}\;\pm \;\text{SE}$	n	$\bar{\text{x}}\;\pm \;\text{SE}$	n	$\bar{\text{x}}\;\pm \;\text{SE}$	n	$\bar{\text{x}}\;\pm \;\text{SE}$
Female adult longevity	19	19.42 ± 1.70a	42	12.45 ± 0.86b	27	8.56 ± 0.60c	26	10.85 ± 0.64b
Male adult longevity	26	20.31 ± 1.14a	33	13.09 ± 0.91b	22	9.50 ± 0.87c	52	8.73 ± 0.61c
Female total lifespan	19	79.53 ± 2.33a	42	44.62 ± 0.84b	27	38.81 ± 0.65d	26	40.77 ± 0.65c
Male total lifespan	26	82.96 ± 1.34a	33	44.03 ± 1.02b	22	39.95 ± 1.09c	52	37.17 ± 0.65d

*n*, effective sample size; $\bar{\text{x}}$, mean value; SE, standard error. Means (± SE) in the same row followed by different letters were significantly different by the pick 1 by 1 test based on the CI of difference (*P*< 0.05).

### Oviposition periods and fecundities

The total pre-oviposition period of females (TPOP) were significantly different except for 25°C and 30°C, with 20°C> 25°C> 30°C> 27°C respectively. There were no significant differences in the adult pre-oviposition periods (APOP) at 20°C, 25°C and 30°C, and the shortest one occurred at 27°C (2.81d). The oviposition periods decreased as temperatures increased from 6.50 days at 20°C to 2.60 days at 30°C, and showed significant differences at 25°C, 27°C and 30°C. The lowest fecundity was observed at 30°C (18.88 eggs/female) and the highest one was observed at 27°C (117.48 eggs/female), with 27°C> 25°C> 20°C> 30°C, respectively (Table 4).

**Table 4:** Female oviposition periods (days) and fecundities (eggs/female) of *Marasmia exigua* at four temperatures.

Item	20°C		25°C		27°C		30°C	
	n	$\bar{\text{x}}\;\pm \;\text{SE}$	n	$\bar{\text{x}}\;\pm \;\text{SE}$	n	$\bar{\text{x}}\;\pm \;\text{SE}$	n	$\bar{\text{x}}\;\pm \;\text{SE}$
TPOP	12	68.25 ± 2.55a	34	37.41 ± 0.50b	21	33.19 ± 0.44c	10	36.10 ± 0.88b
APOP	12	8.42 ± 1.85a	34	5.26 ± 0.46a	21	2.81 ± 0.31b	10	6.50 ± 0.86a
Oviposition days	19	6.50 ± 1.27ab	42	6.44 ± 0.61a	27	4.71 ± 0.57b	26	2.60 ± 0.45c
Fecundity	19	69.95 ± 22.24a	42	111.74 ± 13.02a	27	117.48 ± 17.41a	26	18.88 ± 8.58b

TPOP, the total pre-oviposition period of females (the period counted from egg to her first oviposition); APOP, the adult pre-oviposition periods (the period counted from adult emergence to her first oviposition); *n*, effective sample size;$\bar{x}$, mean value; SE, standard error. Means (± SE) in the same row followed by different letters were significantly different by the pick 1 by 1 test based on the CI of difference (*P*< 0.05).

### Life table analysis

**Fig 2:**
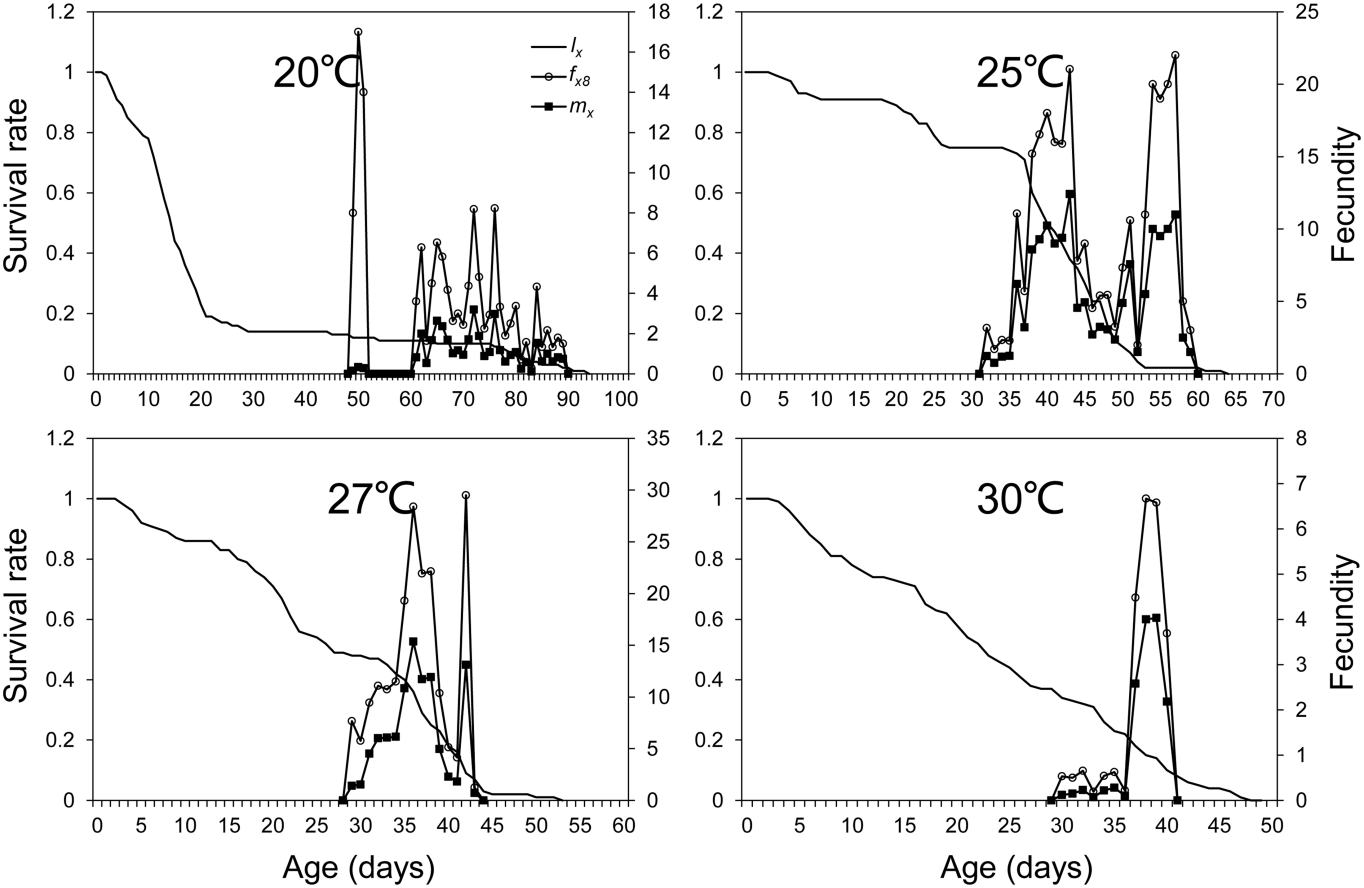
Age-specific survival rate (*l*_*x*_), female fecundity (*f*_*x*8_), and age-specific fecundity (*m*_*x*_) of *Marasmia exigua* at four temperatures.

In our study, most larvae underwent five instars except for a few that extended into a sixth instar stage. The fifth and sixth larvae stages were merged together as L_5-6_. Significant overlaps between stages were observed in the age-stage specific survival rates of *M*. *exigua* at the four temperatures (Fig 1). The stage frequency curves provided descriptions of survival and stage differentiations, while the age-specific survival rate (*l*_*x*_) was a simplified version of the age-stage survival rate and were plotted in Fig 2 together with *f*_*x*8_ and *m*_*x*_ curves. The peak values of *f*_*x*8_ and *m*_*x*_ observed at 27°C were 29.50 on the 42^nd^ day and 15.35 on the 36^th^ day, respectively. Both showed a declining trend with the lowest at 30°C. The reproduction duration decreased from 41 days (at 20°C) to 11 days (at 30°C) as the temperature increased (Fig 2). The intrinsic rate of increase (*r*) observed at 20°C and 30°C showed no significant difference, nor did it at 25°C and 27°C, while *r* at 25°C and 27°C were significantly higher than that at 20°C and 30°C. Similarly the finite rate of increase (*λ*) and net reproduction rate (*R*_*0*_) at 25°C and 27°C were significantly higher than those at 20°C and 30°C. The *r* and *λ* at 27°C were slightly higher than those at 25°C, but the *R*_*0*_ at 27°C (31.10) was lower than that at 25°C (45.56). The mean generation time *T* at the four temperatures showed significant differences with the maximum (70.65d) observed at 20°C and the minimum (35.78d) observed at 27°C, with 20°C> 25°C> 30°C> 27°C in turn (Table 5).

**Table 5:** Population parameters of *Marasmia exigua* at four temperatures.

Temp (°C)	*r*	*λ*	*R*_*0*_	*T*
20	0.017 ± 0.006b	1.017 ± 0.006b	3.32 ± 1.27b	70.65 ± 2.25a
25	0.094 ± 0.005a	1.098 ± 0.005a	45.56 ± 7.57a	40.67 ± 0.65b
27	0.096 ± 0.007a	1.101 ± 0.007a	31.10 ± 6.85a	35.78 ± 0.56d
30	0.020 ± 0.015b	1.020 ± 0.016b	2.17 ± 1.05b	38.41 ± 1.65c

Means (± SE) in the same column followed by different letters were significantly different by using the pick 1 by 1 test based on the CI of difference (*P*< 0.05).

### Lower developmental thresholds and thermal constants

Data were fitted to the linear models describing the relationship between temperature and development rate. The lower developmental threshold temperatures (*t*) and degree day requirements (*K*) of the egg, larva, pr-p, and pre-adult stages of *M*. *exigua* were calculated and are shown in Table 6. The lower development threshold estimated was 12.20°C for the egg stage (20–27°C, *R*^*2*^ = 0.952), 12.80°C for larva (20–27°C, *R*^*2*^ = 0.815), 14.56°C for the pr-p stage (20–27°C, *R*^*2*^ = 0.967) and 11.33°C for the pre-adult stage (20–27°C, *R*^*2*^ = 0.904). And the degree day requirements (*K*) were 66.67, 200.00, 111.11 and 333.33 DD for egg, larva, pr-p, and pre-adult stages respectively (Table 6).

**Table 6:** Lower threshold for development (°C) and thermal requirement (DD) for *Marasmia exigua*.

Stage	Regression equation	*R*^*2*^	*t* (°C) ± SE	*K* (DD) ± SE
Egg	*y* = −0.183 + 0.015*x*	0.952	12.20 ± 0.12	66.67 ± 0.78
Larval	*y* = −0.064 + 0.005*x*	0.815	12.80 ± 0.29	200.00 ± 6.04
Pre-P	*y* = −0.131 + 0.009*x*	0.967	14.56 ± 0.15	111.11 ± 1.57
Pre-adult	*y* = −0.034 + 0.003*x*	0.904	11.33 ± 0.48	333.33 ± 7.22

*R*^*2*^, coefficient of determination.

## Discussion

Understanding the life histories of agricultural pests and their responses to environmental factors is fundamental in the development of pest dynamics and in formulating management strategies [30]. In the past few decades, the ecological characteristics of *M*. *exigua* have been rarely investigated. We present a pioneering attempt to estimate the demography and population parameters of this pest using age-stage, two-sex life tables taking into account the two sexes and stage differentiations and overlaps [31]. Although previous studies had different results from ours [7, 13], the discrepancies might have resulted from factors such as temperature, photoperiod, food, geographic population, number of experimental populations, and population densities [32].

Among the abiotic factors, temperature is the most crucial factor that exerts profound effects on the biology, development, reproduction, distribution, and abundance of insects [33, 34]. In our study, *M*. *exigua* was able to complete development within the temperature range of 20–30°C. The pre-adult development period decreased with an increase in temperature. This might be attributed to the acceleration of metabolism as shown in some other insects such as *Bradysia odoriphaga* [35], *Parapoynx crisonalis* [36], and *Sesamia nonagrioides* [37]. The six larval instars observed at 20°C were different from the five larval instars observed at the other temperatures. The difference between the larval instars in this study might be related to the rearing temperatures. Various factors, like temperature, photoperiod, humidity, and rearing density can affect the number of insect instars [38]. In general, the instar number tends to be higher under adverse conditions. This is consistent with a compensation scenario, in which additional instars might be added when the larvae fail to reach the species-specific threshold size with “normal” numbers of instars under adverse conditions [38]. Similar findings have been reported in other Lepidoptera insects including *Agriphila aeneociliella* [39] and *Brachmia macroscopa* [40]. The relatively higher values of survival rates, fecundities and parameters *r*, *R*_*0*_ at 25 and 27°C might indicate that the temperature range 25–27°C is suitable for the *M*. *exigua* development. A similar optimal temperature has been reported for another species of rice leaffolder, *C*. *medinalis* [41, 42]. The extremely low hatching rates and high mortality rates of *M*. *exigua* at 35°C and15°C demonstrated that the temperatures were unsuitable for development and were close to the insect’s temperature limits. *M*. *exigua* could overwinter as mature larvae in rice stubbles or straw where temperatures are lower than 15°C [10], which likely triggers diapause [43–46]. In addition, the mortalities of egg and the first instar larvae were higher than those of other developmental stages, especially at the unsuitable temperatures (15, 20, and 35°C), indicating that the egg and these stages are relatively more vulnerable.

Large-scale field investigations of *M*. *exigua* in Asia remain scarce. The lower developmental thresholds and thermal constants are useful indicators for geographic distributions and good predictors of life history [29], these two parameters calculated for several temperatures will provide insights into the occurrence possibilities and life history of *M*. *exigua* in specific locations with known temperatures. For example, we might anticipate the occurrence of *M*. *exigua* throughout the year in Southeast Asia, 5 to 6 generations in Fiji, 4 to 5 generations in Southwest China, and 2 to 3 generations in South Korea.

Pest populations are influenced by a variety of biotic and abiotic factors in the field and parameters obtained from laboratory studies need to be taken with caution [31]. However the life table information we obtained can provide a basis for understanding the population ecology of *M*. *exigua* and contribute towards predictions of its population dynamics. In addition to temperature, further studies such as fluctuating temperature, photoperiod and humidity and combined factor effects can supplement our understanding of *M*. *exigua* phenology and thus contribute to the development of management strategies.
